# Access and utilization of healthcare services in Massachusetts, United States: a qualitative study of the perspectives and experiences of Brazilian-born immigrant women

**DOI:** 10.1186/s12913-016-1723-9

**Published:** 2016-09-02

**Authors:** Ana C. Lindsay, Mariana Gonçalves de Oliveira, Sherrie F. Wallington, Mary L. Greaney, Marcia Maria Tavares Machado, Lorita M. Freitag Pagliuca, Carlos Andre Moura Arruda

**Affiliations:** 1 Exercise and Health Sciences Department, College of Nursing and Health Sciences, University of Massachusetts Boston, 100 Morrissey Boulevard, Boston, MA 02125 USA; 2 Department of Nutrition, Harvard School of Public Health, Boston, MA USA; 3 Federal University of Ceara, Nursing School, Fortaleza, Ceara Brazil; 4 Lombardi Comprehensive Cancer Center, Georgetown University Medical Center, Washington, DC USA; 5 Department of Kinesiology, University of Rhode Island, Health Studies, Kingston, RI USA; 6 Department of Public Health, Federal University of Ceara, School of Medicine, Fortaleza, Ceara Brazil

**Keywords:** Access, Brazilian immigrant, Healthcare, Qualitative, Utilization, Women

## Abstract

**Background:**

Understanding immigrants’ interactions with the United States (US) healthcare system will likely make it possible to meet their healthcare needs and improve their quality of life in the US. Although challenges to accessing and utilizing healthcare in the US have been identified, there is little information specific to Brazilian-born immigrants’ experiences. Brazilians comprise a fast-growing immigrant population group in the US. The purpose of this study was to explore Brazilian immigrant women’s perspectives and experiences with healthcare services in the US to gain insights into factors amenable to interventions that may contribute to disparities in access to and utilization of services.

**Methods:**

Five focus groups were conducted from April to May in 2015 using a purposeful sampling of Brazilian-born immigrant women living in Massachusetts, US.

**Results:**

Thirty-five women participated in this study. Although participants expressed their overall satisfaction with the US healthcare system, they noted several barriers to care, including sociocultural differences in delivery of care and communication barriers, including inconsistent quality of interpreting services.

**Conclusions:**

This study provides new information on the experiences and challenges faced by Brazilian immigrant women in accessing and utilizing healthcare services in the US and points out opportunities for improving services and the overall health of this immigrant population. Addressing noted sociocultural differences and communication barriers including inconsistent quality of hospital’s interpreting services might enhance Brazilian-born immigrants’ experiences with the healthcare system.

## Background

One in six residents in the United States (US) is foreign born, and currently 16 % of adults 18+ years of age are immigrants, with the number of immigrants expected to grow to 19 % by the year 2050 [1]. Brazilians began immigrating to the US in increasing numbers in the 1980s due to worsening economic conditions in Brazil [2]. Many of these immigrants are undocumented, and more Brazilian women have immigrated to the US than Brazilian men [2]. The 2006–2010 American Community Survey of approximately 400,000 Brazilian immigrants living in the US determined that nearly half of respondents lived in the northeastern part of the country, primarily in Massachusetts, New York, and New Jersey [2].

Accessing and utilizing health care in a new country is central to the quality of life of immigrants [3–5], yet immigrants often have lower rates of health insurance coverage and medical service usage than their US-born counterparts and immigrant groups with legal status residing in the US [6–10], which impacts their healthcare access. Moreover, immigrants face many challenges accessing healthcare, such as lack of health insurance, financial difficulties, language barriers and lack of interpreters, cultural differences in views about health, health literacy, differences in health expectations, and discrimination based on race or accent [11–16]. Additionally, research suggests that having medical insurance coverage—whether private or through Medicaid—increases utilization of healthcare services by undocumented immigrants [17–19].

In 2010, the Patient Protection and Affordable Care Act, commonly referred to as the Affordable Care Act (ACA) was implemented in the US, to increase the quality and affordability of health insurance and lower the number of uninsured individuals [17, 18]. The ACA explicitly excludes undocumented immigrants, and only requires providing coverage for US citizens and certain documented immigrants [17, 19]. The ACA was in part, modeled on the experience of Massachusetts, the setting for the present study. In 2006, Massachusetts became the first state in the nation to pass comprehensive health reform to provide coverage to the majority of its residents and contain health care costs [18]. Different from the ACA, the Massachusetts healthcare reform, known as Chapter 58, has provisions to offer healthcare coverage to immigrants of different citizenship statuses, who can apply for and receive state-funded health coverage [19]. However, the implementation of the federally mandated ACA in Massachusetts is changing how individuals of all citizenship statuses, especially those with subsidized plans, apply for healthcare coverage [19].

Although a number of studies have identified challenges immigrants face in accessing and utilizing healthcare in their new countries [11, 13–16, 20], there is little information specific to Brazilian-born immigrants’ experiences with healthcare in the US. This information is needed due to differences in sociocultural and contextual circumstances across immigrant groups. Understanding the experiences and challenges faced by specific immigrant groups will likely make it possible to meet their specific needs, and ultimately improve their health status. Therefore, the purpose of this study was to gain an understanding of Brazilian immigrants’ perspectives and experiences with healthcare services in the US in order to provide insights into factors that may contribute to disparities in access to and utilization of services.

## Methods

### Setting

This study was conducted in two cities in Massachusetts, Somerville and Brighton. In Massachusetts, immigrants comprise 15 % of the state’s population. Approximately 65,000 Brazilians, about 19 % of the US national Brazilian population, live in Massachusetts [20].

### Study design and sample

The present study was part of an ongoing community-based, mixed-methods research being conducted among Brazilian families living in the greater Boston area designed to assess Brazilian immigrant mothers’ child feeding and physical activity practices related to the risk of childhood obesity. We worked with predominantly Brazilian churches in the Greater Boston area to recruit Brazilian families to participate in the research project. This current qualitative study was designed to explore Brazilian immigrant women’s perspectives and experiences accessing and utilizing healthcare services and more specifically to: (1) gain an in-depth understanding of immigrant women’s perspectives on healthcare services, and (2) explore how their culture may influence their utilization of and satisfaction with healthcare services. We chose to conduct focus groups (FGs) because they are an important technique for working in diverse cultural settings and provide rich and valuable information [21, 22]. Moreover, the synergistic effects of group settings elicit ideas and discussion that may not arise in individual interviews [21, 22].

### Ethics, consent, and permissions

The Institutional Review Board for the Protection of Human Subjects at the University of Massachusetts–Boston approved the study’s research protocol. Individuals were eligible to participate in the study if they were female Brazilian immigrants, 18+ years of age, and had been living in the US for 12+ months. Due to the present study being part of a larger research project focusing on Brazilian families with young children, most participants were mother of reproductive age. Prior to participating, all individuals received a letter describing the study purpose and procedures, and that participation was voluntary. The letter also stated that participant’s contributions would be unidentifiable in the final report. Participants received a $15 gift card for participating, and childcare was available.

### Data collection

A convenience sample of 35 Brazilian-born immigrant women residing in two cities in the greater Boston area (Somerville and Brighton) participated in the FG discussions. Women were purposively recruited through posted flyers at churches and church events and contacted by research team members during the months of February and March of 2015.

All FGs were held at two local churches between April and May of 2015 and had between five to seven participants per group. FG discussions were conducted in Portuguese and moderated by one of the authors (ACL), a native Portuguese speaker trained in qualitative research methods, using a semi-structured discussion guide. The guide explored four domains specific to healthcare services in the US: (1) access to services, (2) satisfaction with services; (3) barriers faced in accessing and utilizing services; and (4) suggestions for improving services. The discussion guide used open-ended questions and probes and had been pilot-tested in a FG with Brazilian immigrant women and refined prior to use.

Before each FG, the moderator explained in Portuguese the purpose of the study, FG procedures, study confidentiality, and obtained written informed consent from all participants. All FGs, which lasted between 60 and 90 min, were audiotaped. A trained, bilingual (Portuguese and English) research assistant took notes during each FG session. After the discussions, participants completed a self-administered questionnaire that assessed participants’ socio-demographics (e.g., education, marital status, country of origin, and length of time living in the US) and level of acculturation via the Short Acculturation Scale for Hispanics (SASH) [23, 24]. The SASH is a 12-item measuring scale validated for use in Latino groups and was adapted through back-translation to use in Brazilians. The SASH assesses language use, media use, and ethnic social relations [23, 24]. After each FG, the moderator and research assistant convened for 15 min in a private room to discuss new or recurring themes that emerged.

### Analysis

Audiotapes were transcribed verbatim in Portuguese and transcripts were analyzed using thematic analysis, an iterative process of coding data in phases to create meaningful patterns [22, 25, 26]. Analytic phases included: becoming familiar with the data, generating initial codes, searching for and review of themes and patterns, and defining and naming themes [25, 26]. Two authors (ACL, CAMA) read each transcript a number of times to become familiar with the content and then generated initial codes. Items describing a similar idea were grouped, coded manually, and sorted to capture common themes [26, 27]. They next reviewed, defined, named, and identified themes [26, 27]. The same two authors conducted all analyses and resolved discrepancies through discussion. Descriptive statistics were calculated for the sociodemographic data using Microsoft Excel 2008®.

## Results

Saturation was reached with five focus groups, with no new information emerging. Each focus group, on average, had six (range: 5–7) participants.

### Participant characteristics

Participants’ ages ranged from 26 to 56 years (mean = 36.7. years, standard deviation [SD] = 3.6). Approximately 76 % of participants had a high school education, 25 % had completed college, 83 % were married, 89 % were undocumented residents in the US, and 2 % were currently unemployed. The majority (92 %; n = 21) reported being self-employed and owning their own housecleaning business. Approximately half (82 %) reported a family income of $40,000 or less. Women had, on average, two children (range = 1–4). In addition, most spoke Portuguese at home (92 %), watched television programs in Portuguese (95 %), and reported that the majority of their friends were Brazilians (87 %). Participants were originally from various regions of Brazil, including the southeast (e.g., Rio de Janeiro, Espirito Santo, Sao Paulo, and Minas Gerais), the south (e.g., Santa Catarina, Parana, and Rio Grande do Sul), the mid-west (e.g., Goias and Mato Grosso), and the northeast (e.g., Pernanbuco). The length of time that participants had been living in the US ranged from 2 to 20 years (mean = 9 years; standard deviation = 3.4 years).

### Domains and categories

Themes identified within each domain of inquiry are presented below with illustrative quotations.

### Access to healthcare services

#### Healthcare in the US is easily accessible

The majority of women reported being able to easily access healthcare services for themselves and family members in the US. One participant mentioned, “*I have never had any problems with having access to healthcare services since I immigrated to the US,*” Similarly, another participant commented, “*Any time me or one of my family members needs to go to the hospital, we are always seen by the doctor. I never had a problem.”*

Furthermore, several participants reported that it was easier and quicker to access to healthcare services in the US than in Brazil. One participant explained, “*Here, you go to the hospital, they see you right away. They [healthcare professionals] first treat you for whatever you need, and then they ask you if you have health insurance. Back in Brazil, I used to go to the hospital and wait for hours to be seen. And if you have health insurance, it still can take you a long time to be able to make an appointment. You need to be dying for them [healthcare professionals] to see you there [Brazil]*.”

Most participants reported having health insurance, with the majority having “free care” through the state government. Only a few participants reported having private healthcare insurance through work. One woman said, “*I have free care and I never have problem scheduling an appointment to see my doctor. If I need, I can also go to the emergency care, but I prefer not to, I like to see my own doctor.*” Another participant noted, “*I have health insurance through the company I work for, and I have never had any problem being seen by a doctor when I go to the hospital. They treat me very well.*”

#### Confusion with new healthcare regulations

Despite most participants reporting that they had “free care,” some participants reported confusion about options for new healthcare coverage options available under the ACA. Additionally, a few women reported that they had experienced difficulties renewing their healthcare insurance coverage through the State Free Care program, known as “MassHealth.” One participant remarked, “*I always had ‘free care,’ but now they are having some changes in the health insurance, and I am concerned if I will be able to keep my health insurance. A friend of mine told me she was asked to present paperwork to renew her free care.*”

#### Concerns as to whether new healthcare regulations will reduce access

Some participants expressed concerns about whether their healthcare plans offered through Massachusetts’ healthcare plan would change once their plans were renewed. Several participants discussed concerns about not being able to enroll in an insurance plan and losing access to health care. One participant said, “*I am very confused with the new options for healthcare plans. I pay taxes because I hope one day I can get my green card, so I don’t know if I will have to pay for health insurance now, and I really don’t understand all the different options.*”

Furthermore, a few participants reported paying for health insurance out-of-pocket and were concerned about not being able to afford paying for healthcare or increases in healthcare premiums. One participant reported, “*Depending on the family’s salary, they [state health care] are now denying the free healthcare insurance. This happened to me, and I had to have health insurance through my work, and I am paying a lot. It’s expensive, and this is not happening only to me, it’s also happening to other people as well, and will happen to many others.*”

### Satisfaction with healthcare services

#### High levels of satisfaction with US healthcare

The majority of participants reported that their healthcare needs were met in the US, and that they were treated well by healthcare professionals, and satisfied with available healthcare services. One participant remarked, “*I've had various health problems since I moved to the US, and thank God, I only have good things to say about the health care I received in this country. The doctors even call you at home to see how you are doing.*”

#### Prompt access to health services in the US

The main reasons reported for being satisfied with healthcare services were having prompt access to healthcare when needed, and short wait times for scheduled appointments. As one participant noted, “*Here, you are seen right away; any time I needed to see a doctor for me or my children, I had no problems. When you are sick, the last thing you want is to have to wait.*”

In contrast, participants reported difficulty accessing care and long waits to schedule appointments as the main reasons they were dissatisfied with healthcare services in Brazil. One participant explained, “*You wait for hours in long lines, not even knowing if you are going to be seen by a doctor [in Brazil].*”

### Barriers to accessing and utilizing healthcare services in the US

Despite overall satisfaction with healthcare services available in the US, several barriers to using healthcare services in this county emerged.

#### Communication barriers

The majority of participants reported that not being able to communicate directly with healthcare professionals in Portuguese was the main barrier affecting their experiences with the US healthcare system. One participant said, “*When you are not in your own country speaking in your own language, it’s always difficult to understand everything that the doctor is telling you. Even if you can speak some English, it’s not the same as communicating in your language. So, sometimes you are not sure you really understood everything.*” Furthermore, several participants mentioned that not speaking English was an obstacle to accessing and utilizing healthcare services*.* One participant said, “*If you don’t speak English, you have no option; you need to ask for an interpreter. And, it’s not the same as communicating directly with your doctor.*” Another participant added, “*But it is the only option; we are not in our country, and we don’t speak the language, so we need to rely on someone to communicate for us.*”

#### Variation in hospitals’ interpreting services

Participants reported varying levels of satisfaction with the interpreting services offered by hospitals. One participant stated, “*I don’t have any complaints about the interpreting service in the hospital. They are always friendly and helpful.*” However, another woman said, “*Sometimes if you say that you need interpreting services, you need to wait for a long time because they don’t have many interpreters. And the interpreters sometimes are stressed out, and some can be rude. You are trying to explain to them how you are feeling and they keep interrupting you and saying, ‘Just say what’s wrong, what brought you to the hospital.*” Another participant added, “*That’s right. Sometimes you can’t say in a few words, you need to explain the details, but he [interpreter] gets impatient, and you have to rush.*”

#### Cultural differences in healthcare delivery

Another obstacle that affected participants’ healthcare experiences in the US was the perceived cultural differences in approaches to healthcare delivery. These differences were viewed as contributing to misunderstandings of diagnoses and a lack of confidence in treatments prescribed by doctors. One participant mentioned, “*You never leave the hospital knowing what is really causing your pain, what is the diagnosis, and what is causing the pain. So then, they only give you some palliative treatment and send you home.*” Another participant said, “*Also, here in the US, the service is very good, it is excellent, but the quality of prescription of treatment sometimes is not good. I see many people here complaining how much they suffer with not getting the right treatment right away. Sometimes you have to go through many different treatments before finding a solution to their health problem.*”

#### Unmet expectations related to labor and childbirth practices

Across all FGs, participants discussed perceived cultural differences in labor and childbirth practices between Brazil and the US. Participants spoke of their expectations for delivery not always being met in the US with several women reporting they expecting to be admitted to the hospital earlier when having a child. One participant remarked, “*I find the monitoring of pregnancy very good, the prenatal care services are excellent, they offer courses and teach you a lot about how to care for your baby, especially if it is your first baby. Now, when it comes time for childbirth, it’s really hard, you know. The doctors like to keep the mother at home, and they monitor the progress of childbirth over the telephone.*” Another participant added*,* “*I agree, it’s very stressful. You keep calling [the doctor] every five minutes to tell how you are doing. I think it is nerve-wracking. The mother is home and getting apprehensive that something can happen. They don’t like to admit you until it is very close to the baby’s delivery.*”

#### Perceived discrimination by healthcare staff

A few participants reported hostile encounters with and discrimination from healthcare staff when trying to access healthcare services, including a lack of cultural understanding and/or acceptance of cultural differences when assisting immigrant patients. One participant stated, “*It happened to me too. I don’t speak English, you know. I went to the hospital in Everett and wanted to make an appointment to see a doctor, and the receptionist was really rude. I did not understand what she said, so I told in my broken English, ‘I don’t speak English.’ There was a Hispanic patient waiting who said that the receptionist is saying, ‘These people don’t speak English, they should just take a plane and go back to their home country.*”

### Suggestions for improving immigrants' experience with US healthcare services

As discussed below, participants’ suggestions for improvement of healthcare services in the US focused on increasing the availability of services, improving the quality of interpreting services, increasing healthcare professionals’ understanding of cultural differences, and limiting the role of medical students in the delivery of health care.

#### Increasing the availability and improving the quality of interpreting services

As noted earlier, many participants reported communication barriers with interpreting services and inconsistent quality of interpreting services as barriers to treatment and access. One participant explained, “*If you don’t know the language, you have no option, you need to use an interpreter. The problem is that some interpreters don’t have the professional training that they should to know that they should be there to really translate all that you are saying, not to be giving their opinions and telling you [patient] what to say or what not to say.*”

#### Increased understanding of cultural perspectives related to labor and childbirth

Participants compared labor and childbirth practices in the US and Brazil, and nearly all women who had given birth in the US agreed that US healthcare professionals would benefit from a greater understanding of Brazilian women’s perspectives and expectations of labor and childbirth practices. For example, several participants cited long hours of labor and delayed use of anesthetics in the US compared to Brazil. Participants believed that if US healthcare professionals understood this difference and others it would improve patient-provider communication related to the delivery of care during labor and childbirth, and consequently increase patient satisfaction. One participant noted, “*It’s very different than in Brazil; here they make you wait until the last minute to give you anesthesia. You have to beg for it. You are in pain and they make you wait. The doctor wants you [women] to go through labor as if you were in a place without any resources. It can be traumatizing.*”

#### Limiting medical students’ role in delivery of care

Some participants suggested limiting medical students’ roles from being actively involved in delivery of care to observation during consultations and hospital procedures because they were not confident about services rendered directly by medical students. They suggested that doctors should always be present during consultation and treatment. One participant said, “*I don’t mind if the student comes in with the doctor. I know they have to learn, but I do not trust them to do the work of the doctor. I don’t feel safe, so I prefer the doctor, who has more experience, you know?*”

## Discussion

This study used qualitative methodology to explore Brazilian-born immigrant women’s perspectives and experiences accessing and utilizing healthcare services in the US to gain insights into their personal experiences and perceptions of the US healthcare system. The study design allowed for the identification of multiple contextual factors that may influence the experiences of Brazilian immigrant women who access and utilize healthcare services in the US.

The study was conducted in Massachusetts where approximately 15 % of the state’s population is immigrants, of whom approximately 2.5 % are undocumented. The Brazilian immigrant women in this study, almost 90 % of whom, were undocumented, reported easy access to healthcare services in the US and viewed available care as being high quality. This finding differs from that reported by several studies that have found this population group has limited access to healthcare and low levels of satisfaction with services received [3, 11]. Our findings may be a reflection of the study setting and/or study sample. Massachusetts was the first state in the country to pass comprehensive healthcare reform that provides coverage to most of its residents [19]. However, the implementation of the ACA in Massachusetts is changing how residents of various citizenship statuses apply and receive healthcare coverage. In fact, the present study revealed confusion with new insurance options and requirements and concerns about the lack of affordability of increasing healthcare premiums. These findings agree with other studies showing that the general population does not understand new regulations and fears not being able to afford healthcare [28–31]. Therefore, additional education or outreach efforts may be needed. Moreover, participants’ reports of easy access to healthcare may be due to the study’s sample being comprised primarily of women of reproductive age. Women may have more frequent contact with healthcare services than men due to reproductive and maternity needs [6, 8, 13, 32, 33]. Nonetheless, like previous research [5–10], our findings reveal that language difference was an important barrier to utilization of and satisfaction with healthcare services in the US. Being unable to communicate directly with healthcare professionals was a frustrating experience to many women in this study who relied on interpreters.

Our findings revealed that a few women perceived discrimination from some healthcare staff because they could not speak English. Some reported hostile encounters with healthcare staff and a lack of cultural sensitivity and understanding of cultural differences when assisting immigrant patients. This finding agrees with previous studies that have shown perceived discrimination as a barrier to immigrant populations accessing, utilizing, and being satisfied with healthcare services [15, 29, 30, 34].

Like previous studies [8, 12, 35, 36], our study revealed cultural differences in expectations of healthcare practices. These differences were particularly evident in reproductive and maternity healthcare services, and included cultural differences related to doctors decisions regarding labor and delivery in the US. Participants’ perspectives on cultural differences and unmet expectations related to labor and childbirth practices in the US may be due to differences in obstetric health care practices in these two countries. For example, rates of cesarean births in Brazil are high, ranging from 35 to 45 % in the public sector and 80–90 % in the private sector [37, 38]. However, our findings suggest immigrant pregnant women may need additional education and information about differences in childbirth practices and that healthcare professionals may need additional training on cultural issues, differences, and expectations that may impact perceptions of and satisfaction with healthcare services for immigrant populations.

Study findings should be viewed in light of limitations, including limited generalizability due to purposeful sampling and a relatively small sample size. Furthermore, women who chose to participate could have had a heightened interest and awareness regarding the topics. Future research can address these limitations by exploring Brazilian immigrant women’s perspectives and experiences with healthcare from other communities across the US. In addition, women participating in the study were recruited from churches and may already have some community connections, and possibly because of these connections, easier access to health care services. Future studies, should consider recruiting from other community-based settings. Similarly, it is worth noting that men may have very different perspectives and experiences that are important to investigate as well. Study findings should also be interpreted in the context of the study setting. As previously mentioned, Massachusetts is unique in that it offers healthcare coverage to its residents based on residency, and not legal status. Therefore, study findings may not reflect the experience of other undocumented Brazilian immigrants living in other parts of the US. Nonetheless, this qualitative study provides deeper insight into Massachusetts’ population of undocumented Brazilian immigrant women’s personal perceptions and experiences with healthcare services in the US and indicates, importantly, that when given access to appropriate healthcare services, undocumented immigrants utilize it and are satisfied with the health care received.

## Conclusions

Study findings show that undocumented Brazilian immigrant women are satisfied with their access to healthcare services in a state that offers near-universal healthcare coverage. Healthcare is central to quality of life, yet many immigrants and ethnic minorities often lack appropriate access to healthcare and have increased health risks. This study adds to the current literature on accessing and utilizing healthcare services by immigrants living in the US, and also provides new information on Brazilian immigrant women’s perspectives and experiences accessing and utilizing healthcare services in Massachusetts, US, while identifying opportunities for improving services and the overall health of this immigrant population.

## Abbreviations

ACA: Affordable care act
FG: Focus group
LPRs: Legal permanent residents
PPACA: Patient protection and affordable care act
US: United States
